# Assay Format as a Critical Success Factor for Identification of Novel Inhibitor Chemotypes of Tissue-Nonspecific Alkaline Phosphatase from High-Throughput Screening

**DOI:** 10.3390/molecules15053010

**Published:** 2010-04-27

**Authors:** Thomas D.Y. Chung, Eduard Sergienko, José Luis Millán

**Affiliations:** 1 Conrad Prebys Center for Chemical Genomics, Sanford-Burnham Medical Research Institute, La Jolla, CA 92037, USA; E-Mails: tchung@sanfordburnham.org (T.D.Y.C.); esergien@sanfordburnham.org (E.S.); 2 Sanford Children’s Health Research Center, Sanford-Burnham Medical Research Institute, La Jolla, CA 92037, USA

**Keywords:** diethanolamine (DEA), absorption spectroscopy, luminescence, high throughput screening, CDP-Star®, Molecular Libraries, tissue-nonspecific alkaline phosphatase, alkaline phosphatase, chemical library, *para*-nitrophenylphosphate

## Abstract

The tissue-nonspecific alkaline phosphatase (TNAP) isozyme is centrally involved in the control of normal skeletal mineralization and pathophysiological abnormalities that lead to disease states such as hypophosphatasia, osteoarthritis, ankylosis and vascular calcification. TNAP acts in concert with the nucleoside triphosphate pyrophosphohydrolase-1 (NPP1) and the Ankylosis protein to regulate the extracellular concentrations of inorganic pyrophosphate (PP_i_), a potent inhibitor of mineralization. In this review we describe the serial development of two miniaturized high-throughput screens (HTS) for TNAP inhibitors that differ in both signal generation and detection formats, but more critically in the concentrations of a terminal alcohol acceptor used. These assay improvements allowed the rescue of the initially unsuccessful screening campaign against a large small molecule chemical library, but moreover enabled the discovery of several unique classes of molecules with distinct mechanisms of action and selectivity against the related placental (PLAP) and intestinal (IAP) alkaline phosphatase isozymes. This illustrates the underappreciated impact of the underlying fundamental assay configuration on screening success, beyond mere signal generation and detection formats.

## 1. Introduction

Alkaline phosphatases (E.C.3.1.3.1) (APs) are dimeric enzymes, present in most organisms [1] where they catalyze the hydrolysis of phosphomonoesters. In humans, there are three tissue-specific isozymes: intestinal (IAP), placental (PLAP), and germ cell (GCAP) APs. A fourth AP is tissue-nonspecific (TNAP) and is expressed at high levels in bone, liver, and kidney [2]. TNAP is centrally involved in mechanisms that control normal skeletal mineralization and pathophysiological abnormalities that lead to disease states such as hypophosphatasia, osteoarthritis, ankylosis and vascular calcification [2]. TNAP acts in concert with the nucleosidetriphosphate pyrophosphohydrolase-1 (NPP1) and the Ankylosis protein to regulate the extracellular concentrations of inorganic pyrophosphate (PP_i_), a potent inhibitor of hydroxyapatite formation at concentrations normally found in plasma [3,4,5]. 

Mutations in the TNAP gene (*ALPL*) leads to hypophosphatasia, an inborn error of metabolism that features rickets or osteomalacia as a result of accumulated levels of extracellular PP_i_, resulting from suboptimal TNAP’s pyrophosphatase activity [6,7,8]. Conversely, mutations in the NPP1 [9] or ANK gene [10], lead to soft-tissue calcification, including vascular calcification, as a result of reduced production of transport of PP_i_ [11]. Normalization of ePP_i_ levels in NPP1 null and ANK-deficient mice improves their soft-tissue ossification abnormalities [12,13]. Our studies have revealed that crossbreeding either the *Enpp1*^−/−^ or the *ank/ank* mice to mice deficient in TNAP (*Akp2*^−/−^) mice normalizes ePP_i_ levels and induces a secondary up-regulation of osteopontin (OPN) levels [14]. Importantly, these studies have suggested that TNAP may be a useful therapeutic target for the treatment of vascular calcification [11]. 

Vascular calcification refers to the deposition of hydroxyapatite in cardiovascular tissues, such as arteries and heart valves, and is a significant risk factor for cardiovascular disease and death [15]. Intimal calcification is a sequella of atherosclerosis associated with inflammation. Medial calcification is independent of atherosclerosis and inflammation, and occurs in renal failure, diabetes, obesity and aging, where it is thought to increase morbidity and mortality through decreased arterial compliance. Humans undergoing chronic hemodialysis have reduced plasma levels of PP_i_ and commonly have arterial calcification [16] and provide compelling data that PP_i_ is an important endogenous mineralization inhibitor of medial vascular calcification. Furthermore, our experimental data have shown that the transgenic overexpression of TNAP in the vascular media leads to calcification [17] and that TNAP activity is upregulated in *Enpp1*^−/−^ and *ank/ank* vascular smooth muscle cells (VSMCs), as well as in the aorta of uremic rats [11,18]. This suggests that upregulation of TNAP activity contributes to PP_i_ deficiency and the ensuing medial calcification.

IAP has long been known to be associated with lipid absorption on the basis of the following evidence: (1) during fat absorption, parallel increases in IAP activity and triacylglycerol concentration are observed in the thoracic duct lymph [19]; (2) IAP is associated with chylomicron secretion [20,21] but not with chylomicron formation [22], and serum IAP levels are correlated with the levels of apolipoprotein B-48, a protein exclusive to intestinal chylomicrons in humans [23]; (3) IAP is found in the membrane surrounding the neutral fat droplets in the villi of the intestinal mucosa during fat absorption [20,21] and is thought to transport dietary lipids from the intestinal tract into the circulation as a component of unilamellar membranes called surfactant-like particles [24]; (4) IAP knockout mice become obese when fed a high fat diet and show an accelerated transport of lipids in the gut [25], which leads to visceral fat accumulation and hepatic steatosis [26]. However, the exact biological function(s) and the mechanism for the involvement of IAP in lipid absorption are still unknown. Furthermore, through its ability to detoxify lipopolysaccharide (LPS), a gram-negative bacteria endotoxin, IAP has been shown to act as a gut mucosal defense factor, maintained by enteral nutrition [27]. PLAP is highly expressed in the placental tissue of primates [2]. Despite numerous clinical studies to evaluate the usefulness of PLAP in pregnancy and as a cancer marker, almost nothing is known about its putative biological function [2]. PLAP has been reported to behave as an Fc receptor during pregnancy [28]. PLAP has also been proposed to act as a fetal growth factor [29]. The identification of PLAP-specific inhibitors with selectivity over TNAP and IAP will provide the necessary tools to help advance studies to elucidate its biological role. Even less is known about the biological function of the highly homologous GCAP isozyme, expressed in testicular germ cells and re-expressed in testicular cancer [2,30]. Given that both PLAP and GCAP are newcomers in the evolutionary scene, just preceding the divergence of Old World Monkeys and Apes, there are no equivalent murine genes to enable knockout studies to investigate their function. Furthermore, to-date there is a paucity of molecular probes, including small molecule compounds, able to specifically discriminate between and specifically interfere with PLAP and GCAP function. 

Finally, we have recently demonstrated shown that increases in tissular and circulating levels of TNAP lead to higher bone mineral density (BMD) by reducing the effective levels of the calcification inhibitors PP_i_ and OPN (Narisawa *et al.* unpublished observations). These data provided a mechanistic interpretation for the correlation between TNAP and bone mineral density that has been observed in humans and mice [31,32]. Furthermore, these studies suggested the possibility that administration of recombinant TNAP itself, or of pharmacological activators of TNAP’s pyrophosphatase activity, may serve as therapeutics drugs for the treatment of hypophosphatasia and/or osteoporosis. Indeed, we have recently shown that bone-targeted TNAP can completely prevent all the manifestations of infantile hypophosphatasia in the TNAP knockout mouse model [33]. The specific aims of this HTS project were to identify small molecule compounds in the NIH Molecular Libraries Small Molecule Repository (MLSMR) that were highly specific activators of TNAP using a luminescence-based assay; test the confirmed positives in a secondary assay with natural substrates of TNAP, check for specificity against other recombinant phosphatases and test confirmed positives for their ability to increase calcification in osteoblast cultures. The novel chemical probes identified in this way might ultimately lead to a novel therapy for hypophosphatasia and/or osteoporosis patients.

**Figure 1:**
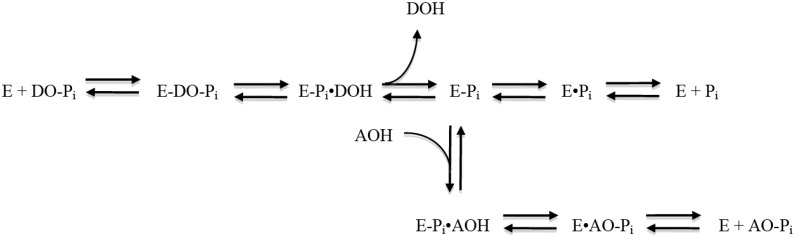
Catalytic mechanism of the alkaline phosphatase reaction (see [34]). E, alkaline phosphatase enzyme molecule; DO-P_i_, substrate molecule; E-P_i_, phosphoenzyme (enzyme phosphorylated on Ser-93 in the TNAP sequence of its active site); DOH, product alcohol; E·P_i_, noncovalent complex of inorganic phosphate in the active site; AOH, an alcohol molecule, acceptor substrate of transphosphorylation reaction acceptor; AO-P_i_, product of transphosphorylation reaction.

The molecular mechanism of the AP catalytic reaction is common to the enzyme from various species and tissues [34] and is depicted in Figure 1. The initial reaction catalyzed by AP (designated as E in the figure) consists of a phosphate donor substrate (DO-Pi) binding step, phosphate-moiety transfer to the active site Ser, and first product alcohol (DOH) release. In the second part of the reaction, the second product phosphate is released through hydrolysis of the covalent intermediate (E-P_i_) and dissociation of inorganic phosphate from the non-covalent complex (E·P_i_). Depending on the origin of the enzyme and the exact conditions of the reaction, either hydrolysis of E-P_i_ or release of the phosphate from E·P_i_ is *rate limiting*, resulting in the enzyme being kinetically “trapped” in these enzyme-P_i_ forms. However, in the presence of some amine-containing alcohols (AOH), phosphate is preferentially released from enzyme via a transphosphorylation reaction, yielding phosphorylated amino alcohols and the regeneration of free enzyme. This path provides >100-fold acceleration to the phosphate release. Thus, amine-containing alcohols serve as the second substrate molecule in the Ping Pong Bi Bi mechanism of AP reaction by substituting for the less efficient substrate water. AP assays commonly used in clinical practice [35] are based on dephosphorylation of *p*-nitrophenol phosphate (pNPP) in the presence of a high concentration of amine-alcohol-containing buffers, such as 2-amino-2-methyl-1-propanol and diethanolamine (DEA). Besides maintaining an alkaline pH, the buffer also provides saturating levels of phosphate acceptor substrate for the AP transphosphorylation reaction necessary for boosting the assay sensitivity. Interestingly, the biological significance of AOH is still unknown, and it is unclear if any biological molecule is capable of performing the function of AOH. 

TNAP, as all mammalian APs, is inhibited uncompetitively by a limited number of inhibitors (see [2] for complete review). Uncompetitive inhibitors bind only to the enzyme-substrate (ES) complex and therefore cannot be overcome by high substrate concentrations and indeed work best when enzyme is saturated with the substrate. They include L-homoarginine [36] as well as some unrelated compounds, such as levamisole [37] and theophylline [38]. However, these known inhibitors of TNAP are not entirely specific for this AP isozyme, have very low affinity (in the millimolar range) and are weak inhibitors of the pyrophosphatase activity of TNAP. For these reasons, we recently initiated a comprehensive and ambitious program to develop new potent and selective small molecules capable of modulating the pyrophosphatase activity of TNAP at physiological pH and at concentrations attainable *in vivo*. We started by characterizing the precise active site pocket that accommodates the known TNAP inhibitors by comparing the three-dimensional structures of TNAP and PLAP - isozymes that differ significantly in inhibitor specificity, and then using site-directed mutagenesis to substitute TNAP residues to their respective homologues in PLAP [39,40]. We found two distinct areas in the TNAP active site able to accommodate inhibitors; the first, comprising residues R433 and H434, accommodates hydrophobic ringed structures such as levamisole and theophylline, while the second, comprising residues E108/G109 can accommodate more hydrophilic extended inhibitors such a L-homoarginine [2,40]. 

In this article we describe the serial development of two miniaturized high-throughput screens (HTS) for TNAP inhibitors that differ in both signal generation and detection formats, but more critically in the concentrations of a terminal alcohol acceptor used. These assay improvements allowed the rescue of the initially unsuccessful screening campaign against a large small molecule chemical library, but moreover enabled the discovery of several unique classes of molecules with distinct mechanisms of action and selectivity against the related PLAP and IAP isozymes. This illustrates the underappreciated impact of the underlying fundamental assay configuration on screening success, beyond mere signal generation and detection formats. These studies have clear translational implications. Potent pyrophosphatase inhibitors would be useful for the treatment of medial vascular calcification, as well as soft-tissue calcifications associated with NPP1 and ANK deficiencies. Additionally, the increased sensitivity, expanded dynamic range of the improved assay allowed it to also be used the find small molecule activators. Activators of TNAP function may be useful for the treatment of milder forms of hypophosphatasia, as the therapeutic use of TNAP activators might sufficiently enhance residual TNAP activity to help resolve or prevent the rickets/osteomalacia characteristic of the disease and may also be useful as a means of increasing bone mineral density in patients with osteoporosis.

## 2. Results and Discussion

### 2.1. Results of HTS Using the Colorimetric Assay

The screen for TNAP inhibitors was brought into the MLSCN from an X01 grant. The assay employed a variation of a clinical AP test [35] that is based on a colorimetric detection of AP activity using pNPP as the chromogenic substrate in the presence of a saturating concentration of phosphate acceptor substrate DEA. This assay was used to screen the ChemBridge DIVERSet™ and identified three compounds, MLS-5361418, MLS-5923412 and MLS-5804079 (Table 1), in addition to levamisole which had been previously reported by the one of us to act as an inhibitor of TNAP with a K_i_ = 21.4 µM [11].

These compounds were of modest potency, but did show efficacy in *ex vivo* models of calcification to establish proof-of-concept [11]. It was therefore proposed to use this assay to screen the entire then ~64,000 member MLSMR library (during the pilot phase of the NIH Roadmap Initiative). We noted however that, while the assay uses fairly low cost reagents, due to the relatively low absorption coefficient of the pNPP substrate the dynamic range of the assay is relatively low and higher amounts of TNAP are required. Also, the high ionic strength of the reaction buffer and potential color interference (405 nm E_max_) of yellow-colored compounds shed some concern over the assay performance and sensitivity. Therefore two additional assays were developed at our screening center, the *Conrad Prebys* Center for Chemical Genomics (CPCCG). One of them modifies the colorimetric detection of phosphate released from pNPP by using a malachite green-molybdate reagent with a higher extinction coefficient than pNPP, yielding therefore a higher sensitivity. Due to the high buffer capacity of 1M DEA, pH 9.8 present in the colorimetric assay, a specially formulated detection reagent was utilized. The modified assay also ensured optimal sensitivity for detecting ligands with different modes of action (MOA by setting the concentrations of both substrates to their Michaelis constant (K_m_) values. The details of the assay development, validation and HTS are described elsewhere [41,42], where it was found to be a robust and reproducible assay with good Z-factors and clear thresholds during implementation. However, after an initial screen of 20,500 MLSMR compounds using p-nitrophenol detection (see PubChem AID 615) or malachite green–based phosphate detection assay (see PubChem AID 614), to our surprise and consternation no hits were obtained over the background threshold.

**Table 1:** *In vitro* data on existing inhibitors of TNAP.

Compound	Structure	TNAP- Color K_i_ (µM)
Levamisole	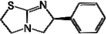	21.4
MLS-5361418	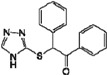	5.6
MLS-5923412	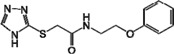	5.6
MLS-5804079	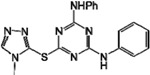	6.5

### 2.2. Pilot Screen of the NIH MLSMR Library with the Luminescent TNAP Assay

In response to this lack of hits, we adapted into a miniaturized plate based assay, a second novel TNAP assay based on CDP-star, a luminescent substrate that had been developed and optimized for the detection of alkaline phosphatases in blotting techniques [43]. In contrast to the colorimetric assay, the luminescent assay is about three orders of magnitude more sensitive than the colorimetric assay. It was optimized to work at a sub-K_m_ concentration of DEA and lower concentration of the enzyme. Following hydrolysis of the CDP-Star substrate, the resultant unstable dioxetane intermediate collapses with a sustained chemiluminescent signal (glow *vs.* flash) that produces maximal light emission at 60 minutes that continues for up to 25 hours (Sigma Prod. No. U-ALK information), requires no quenching and, therefore, provides an automation-friendly assay. 

A detailed description of the development and utilization of the novel luminescent HTS assay for TNAP is described elsewhere [41,42]. Briefly, the assay was optimized to enable screening TNAP in the presence of phosphate-donor and phosphate-acceptor substrates present at their respective K_m_ values. TNAP was screened against the MLSMR collection containing 64,394 compounds. The average Z′ factor for the full screen was equal to 0.82 (range, 0.75–0.89), and every plate was therefore acceptable. 

### 2.3. Comparison of the Pilot Screen with the Colorimetric and the Luminescent TNAP Assay

**Figure 2:**
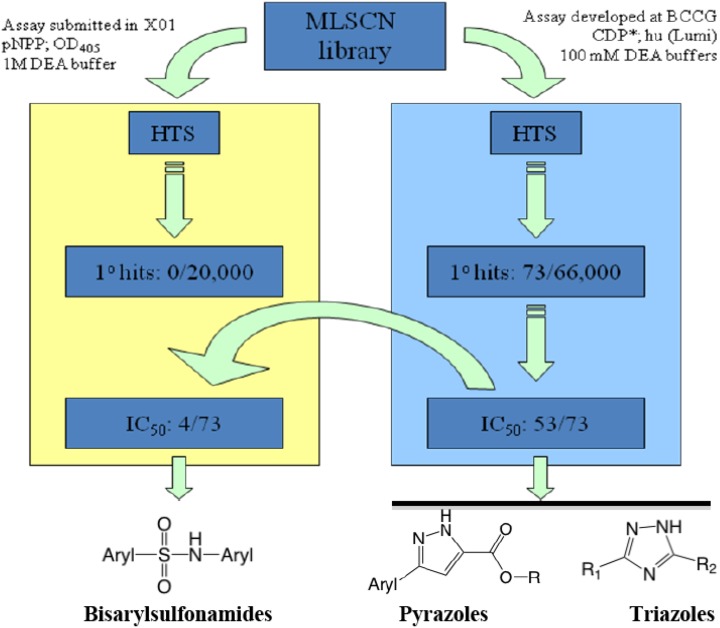
Comparison of the TNAP inhibitors screen in the colorimetric and luminescent formats.

Figure 2 schematically presents the workflow and results from both colorimetric and luminescent HTS screens. From the luminescent primary screen (right hand box), 73 primary positive compounds causing >50% inhibition, were identified (0.11% hit rate). For initial hit confirmation, aliquots for all the primary positives were obtained from the MLSMR, and the compounds were tested in quadruplicate at the concentration used in primary screening. Hits were similarly tested in the two colorimetric assays (left hand box, see Materials and Methods), OD405 (pNPP) and MG (malachite green). Only four of the 73 hits from the luminescent assay were confirmed in either colorimetric assay ([pNPP] or [MG], not shown), whereas 55 of the primary hits were reconfirmed in the primary HTS luminescent assay. This may be rationalized because the TNAP activity with pNPP substrate and the sensitivity of the detection used are quite low at the saturating levels of 1M DEA (roughly 10 × K_m_) used to accelerate the turnover rate. In contrast, the luminescent assay is extremely sensitive and could easily be performed in the absence of DEA; however, for the purposes of this HTS project, the concentration of DEA was kept at its apparent K_m_ value equal to 0.1M [41]. All of the hits that were active in the colorimetric assay belonged to the bisaryl sulfonamide scaffold and were found to be non-competitive with DEA [44]. It is important to emphasize that neither the pyrazole nor triazole scaffolds would have been identified by the colorimetric assay.

To test this hypothesis for the apparent discrepancy in hit confirmation, we also tested the 73 active compounds in the luminescent assay, performed in the presence of 1M DEA-based buffers similar to the colorimetric assays. The number of hits significantly decreased and matched the hit rate of the colorimetric assays, suggesting that a high DEA concentration interferes with the binding of most of the hits, perhaps through a direct competition for the binding site. Full dose-response analysis of 73 primary HTS hits was performed with the luminescent-based assay at 0.1M DEA, and 53 compounds were confirmed as hits that gave IC_50_ values below 20 μM ranging from micromolar to submicromolar. Two of the compounds were found to inhibit PLAP rather than TNAP and were not followed any further. Protocol and experimental data for TNAP luminescent-based screening are described in PubChem AID 518. The results of the HTS campaign are summarized in Table 2. 

**Table 2:** Summary of High-Throughput Screen for Tissue-Nonspecific Alkaline Phosphatase with the Luminescent Assay.

	Compounds Screened	Hits Identified	Hit Criteria	Hit Rate (%)
**Primary HTS**	64,394	73	>50% Inhibition	0.11
**Duplicate single-concentration confirmation**	73	55	>50% Inhibition	75.3
**Dose-response confirmation**	55	53	IC_50_ < 20 µM	96.4

**Table 3:** Confirmation of Major HTS Scaffolds from Dry Powders.

Compound ID	PubChemCID	Structure	IC_50_ (µM)				
			HTS-Lumi*	HTS-Color*	SAR-Lumi**	SAR-Color**	SAR-MG**
MLS-0010847	645853	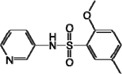	1.8	0.71	0.93	0.83	0.95
MLS-0038949	2931238	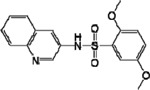	0.16	0.04	0.19	0.116	0.19
MLS-0005718	646303	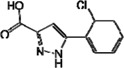	0.98	>100	0.74	>100	>100
MLS-0039961	610864	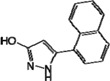	3.2	21	4.6	46	74
MLS-0067142	2975791	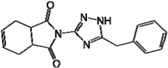	0.24	>100	.19	>100	>100

HTS, high-throughput screening; SAR, structure-activity relationship. * Compounds were obtained from the Molecular Libraries Small Molecule Repository in liquid form. ** Compounds were obtained from corresponding vendors as fresh solid samples.

All 53 compounds identified and confirmed as positive inhibitors of TNAP originated from commercial sources. This significantly simplified compound structure verification and potency confirmation was performed on fresh solid compounds. The representatives of the major scaffolds were purchased from their original vendors. Table 3 summarizes the reconfirmation results for lead compounds of each scaffold, and it is notable that potency values from fresh solids (SAR IC_50_ values in Table 2) closely match those obtained from fresh solutions provided by the MLSMR (HTS IC_50_ values). Extended SAR studies for these scaffolds are described elsewhere [44].

### 2.4. Primary HTS Data Analysis

*Clustering analysis.* The 53 confirmed dose-responsive hit molecules were clustered into four scaffold groups (see Figure 2) and several singletons using Chemistry Component Pipeline Pilot Software (Accelrys, San Diego, CA). Expanded HTS data analysis in relation to the hits was performed on three scaffolds: (1) bisarylsulfonamides, (2) pyrazoles, and (3) triazoles, as noted in Figure 2, each possessing members with submicromolar potency. The representatives of the major scaffolds were purchased from their original vendors. Table 3 summarizes the reconfirmation results for lead compounds of each scaffold, and it is notable that potency values from fresh solids (SAR IC_50_ values in Table 2) closely match those obtained from fresh solutions provided by the MLSMR (HTS IC_50_ values). We purchased and tested 46, 29, and 10 commercially available analogs of the bisaryl sulfonamide, pyrazole, and triazole series, respectively. These analogs offered confirmation for the nascent SAR conclusions drawn from analysis of HTS data.

*Selectivity of Primary Actives Against other Phosphatases*: To assess the selectivity of compounds across the alkaline phosphatase family, luminescent assays were developed for human PLAP and human IAP with the same CDP-Star reagent. As mentioned above, the PLAP selectivity assay (PubChem AID 1512) was implemented as an integral part of the hit optimization process. For lead characterization, we added a luminescent assay for IAP (PubChem AID 1017) to the selectivity panel. The selectivity data for the best representatives of the scaffolds, obtained at our screening center (*CPCCG*) and extracted from the PubChem database, are summarized in Table 4. Bisarylsulfonamide scaffold compounds did not inhibit PLAP or IAP and were inactive against more than 200 assays in PubChem. Both pyrazole compounds did not inhibit PLAP and had reasonable selectivity against IAP, whereas the triazole compound was equipotent against TNAP and IAP although slightly less potent against PLAP.

**Table 4:** Selectivity of Primary TNAP Scaffolds against Other Alkaline Phosphatases.

Compound ID	Structure	Selectivity Panel IC_50_ (µM)			# Assays in PubChem	
		TNAP	PLAP	IAP	Active	Total
MLS-0010847	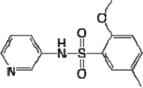	0.93	>100	>100	1 (TNAP)	234
MLS-0038949	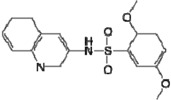	0.19	>100	>100	2 (TNAP, CYP2C19)	214
MLS-0005718	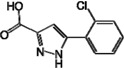	0.74	>100	44	1 (TNAP)	242
MLS-0039961	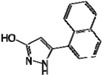	4.3	>100	>100	1 (TNAP)	213
MLS-0067142	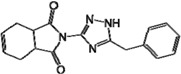	0.19	3.1	0.54	2 (TNAP, IAP)	221

### 2.5. SAR Development and Characterization of Major Scaffolds Classes

*Bisarylsulfonamides series.* For the bisarylsulfonamide scaffold (Figure 2) series, the three most active compounds of the four hits shown in Table 4 share high similarity to each other: all have a 3-amino-substituted pyridine or quinoline moiety and an alkoxy group at the *ortho* position of the sulfonylbenzene. Although the fourth weaker compound lacked the pyridine moiety, it still contained the *o*-alkoxy group. DEA variation studies showed that MLS-0005718 and MLS- 0067142 inhibition was strongly affected by DEA, consistent with a competitive mechanism (Table 5). In contrast, inhibition by MLS-0038949 and MLS-0039961 was virtually identical at all three DEA concentrations tested, suggesting these were noncompetitive inhibitors [41].

**Figure 3:**
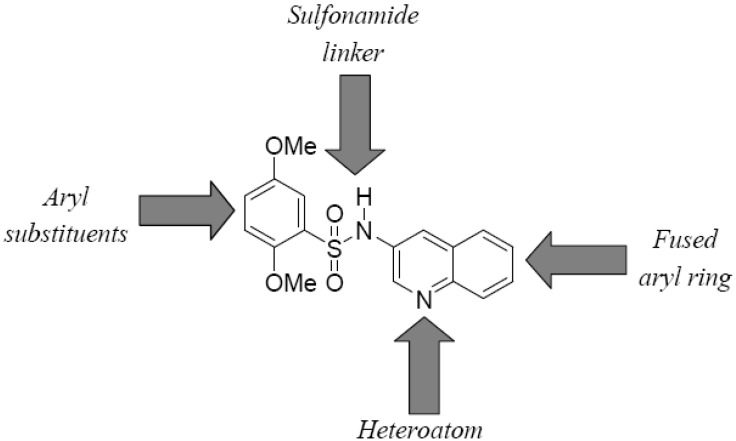
Structural elements for SAR of bisarylsulfonamides.

Four elements in the core scaffold were varied in the SAR exploration (Figure 3): (a) the quinoline-fused aryl ring; (b) the aryl substituents; (c) the sulfonamide linker; and (d) the quinoline heteroatom. Analogs of hits were characterized in the luminescence-based assay (TNAP-L, see Table 3) to determine potency against TNAP and the potency data for selected analogs of help define CID-2931238 are shown in Table 3. Preliminary *in vitro* data for this series suggests that despite the fact that the SAR around the lead structure is tight, there is a tractable SAR emerging nonetheless. To date, 55 analogs of CID-2931238 have been purchased and tested, while a further 61 analogs have been synthesized. In order to evaluate the importance of the fused aryl ring, an analog of CID-2931238 was synthesized and tested in which the quinoline moiety was replaced with pyridine (MLS-0068486). This led to a reduction in potency of roughly 4-fold compared with the lead structure (see Table 5). Preliminary data also suggest that the substituents on the phenyl ring are critical for potency. For example the analog MLS-0090845, in which both methoxy substituents were removed, is inactive in the assay up to 100 µM. Compound MLS-0090844 is an exact analog of the lead structure in which the 2-methoxy substituent is removed, and it is 100-fold less potent. Similarly, the 3,4-dimethoxy- (MLS-0090842) and 2,4-dimethoxy- (MLS-0111875) derivatives were inactive or weakly potent (19.5 µM), respectively. We have also synthesized analogs of the lead structure in which the sulfonamide linker is reversed or replaced with an amide. This includes the –SO_2_NH- to –NHCH_2_- replacement analogs MLS-0111667 and MLS-0111666, and the amide derivative MLS-0111666, all of which were inactive *in vitro.*

**Table 5:** SAR of the bisarylsulfonamide series. 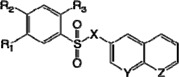

Compound No.	R1	R2	R3	W	X	Y	Z	TNAP-Lumi IC_50_ (µM)
CID-2931238	CH_3_O	H	CH_3_O	SO_2_	NH	N	CH	0.25
MLS-0068486	CH_3_O	H	CH_3_O	SO_2_	NH	N	No ring	0.92
MLS-0090845	H	H	H	SO_2_	NH	N	CH	NA
MLS-0090844	CH_3_O	H	H	SO_2_	NH	N	CH	25.7
MLS-0090842	CH_3_O	CH_3_O	H	SO_2_	NH	N	CH	NA
MLS-0111875	CH_3_O	H	CH_3_O	SO_2_	NH	N	CH	19.5
MLS-0111667	CH_3_O	H	CH_3_O	CH_2_	NH	N	CH	NA
MLS-0111668	CH_3_O	H	CH_3_O	NH	CH2	N	CH	NA
MLS-0111666	CH_3_O	H	CH_3_O	CO	NH	N	CH	NA
MLS-0111639	CH_3_O	H	CH_3_O	SO_2_	NH	CH	N	NA

Finally, the presence and/or position of the quinoline heteroatom is important for the potency of the scaffold. Several analogs were tested in which the quinoline moiety was replaced with naphthyl *i.e.*, substitution of N for C in the molecular framework, and this modification results in complete ablation of *in vitro* activity (data not shown). Similarly, transposition of the quinoline heteroatom, as in MLS-0111639, eliminates activity.

Inorganic pyrophosphate (PP_i_) and pyridoxal-5’-phosphate (PLP), a form of vitamin B6, are the endogenous substrates for TNAP [2]. As with other alkaline phosphatases, the hydrolysis of the phosphoserine intermediate is the rate-limiting step of the TNAP overall reaction and consequently in the steady state the majority of the enzyme exists in the covalent intermediate form. The compound CID-2931238 inhibits TNAP catalytic activity through reversible equilibrium binding. No difference was noticed in compound potency whether TNAP was pre-incubated with the compound or not. The binding of CID-2931238 is uncompetitive *versus* the phosphate donating substrate (CDP-star or pNPP) and non-competitive *versus* the phosphate accepting substrate, such as DEA.

**Figure 4:**
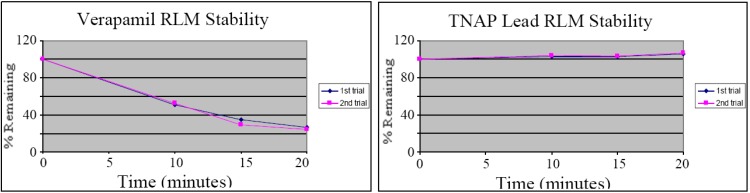
Liver microsome stability of CID-2931238 (duplicate runs).

The compound CID-2931238 demonstrated predictable behavior in the performed TNAP assays. Its average IC_50_ values were equal to 193, 158 and 192 nM in the luminescent, colorimetric *p*-nitrophenol release and colorimetric phosphate-release assays, respectively. The average Hill coefficient values observed in these assays were equal to 1.12, 1.02, and 0.99, respectively. The CID-2931238 compound was soluble in aqueous solutions at concentrations below 25 µM. It had no effect on GAPDH activity and demonstrated no cytotoxicity and is inactive in 131 other assays deposited in PubChem [44]. Indeed compound CID-2931238 was tested in assays against two other alkaline phosphatases, PLAP and IAP, which share 50% and 52%, sequence identity with TNAP, respectively. The compound did not show any inhibition at or below 100 µM in these assays, providing a >500 selectivity index against the closest counter-targets. The compound CID-2931238 appears to be a novel tool for the characterization of TNAP activity in various biological systems. Given the extracellular localization of TNAP, the compound could presumably serve as a useful TNAP chemical probe in animal studies. CID-2931238 was therefore also evaluated in an *in vitro* assay measuring stability in rat liver microsomes (RLM) to provide an assessment of the suitability of the compound for *in vivo* experiments [44]. After a 20-minute incubation virtually all-of-the compound remained, compared to verapamil (see Figure 4), suggesting that CID-2931238 may be suitable for *in vivo* proof-of-concept studies.

**Figure 5:**
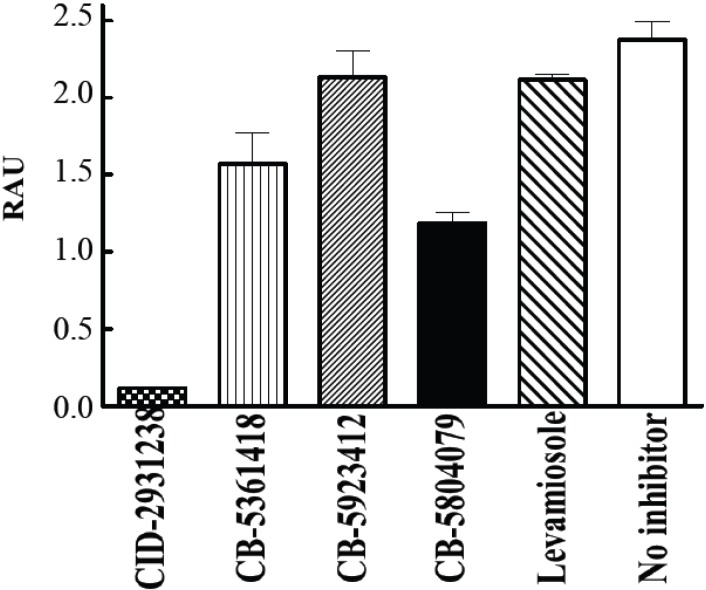
Suppression of calcification by CID-2931238 in primary calvarial osteoblast cultures [11]. Osteoblasts from calvaria bones of WT mice were cultured for 19 days. Media supplemented with 50 µg/mL ascorbic acid and 10 mM β-glycerophosphate were renewed every second or third day (total seven medium changes). The concentration of each compound was maintained at 30 µM. RAU = relative absorbance units.

Indeed CID-2931238 inhibits calcification in primary calvarial osteoblast cultures [11]. Figure 5 shows that CID-2931238 completely inhibited mineralization in this culture system. It appeared much more potent than either levamisole or the ChemBridge compounds discovered from the original colorimetric screen. CID-2931238 exceeds in potency all existing TNAP inhibitors in both *in vitro* and *ex vivo* assays.

*Pyrazole series.* The available HTS data did not suggest immediately which aryl group or R group is preferred for this pyrazole scaffold (Figure 2, Table 2). However, a similar HTS data analysis suggested that a non-substituted position 4 of the pyrazole ring is preferred for TNAP inhibition. Extended SAR studies and medicinal chemistry for these scaffolds are described elsewhere [45] and summarized herein.

**Figure 6:**
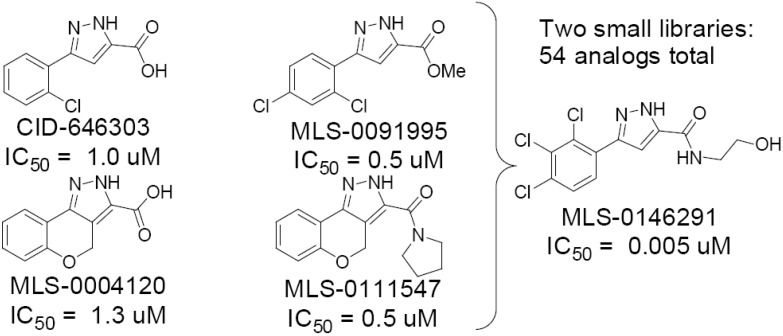
Summary of CID-646303 series optimization.

During the initial phase of testing of purchased analogs of the confirmed pyrazole hit, CID-646303, we discovered some important features of the SAR that improved potency 2-fold in this series from 0.98 µM IC_50_ for the lead to 0.5 µM IC_50_ for the 2,4-dichlorophenyl ester derivative MLS-0091995 (see Figure 6). Furthermore, conversion of the tricyclic derivative MLS-0004120, with an IC_50_ value of 1.33 µM, to the pyrrolidine amide analog MLS-0111547 leads to a 3-fold improvement in potency (0.48 µM IC_50_). We used this information to design a library of amide analogs as shown in Figure 3 and the Schemes below. Two iterations of synthesis and testing of a total of 54 analogs led to the identification of five analogs with potency values in the range of 20–70 nM (see Table 6).

**Table 6:** SAR on selected analogs of the optimized pyrazole series. 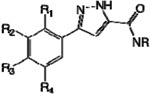

Compound	R1	R2	R3	R4	RNH_2_	TNAP Lumi IC_50_ (µM)
MLS-0146161	Cl	H	Cl	H	NH_2_CH_2_CH_2_OH	0.020
MLS-0146149	Cl	H	Cl	H	NH_2_CH(CH_3_)_2_	0.024
MLS-0146165	Cl	H	Cl	F	NH_2_CH_2_CH_2_OH	0.042
MLS-0146145	Cl	H	Cl	H	NHNH_2_	0.052
MLS-0146148	Cl	H	Cl	H	N(CH_3_)_2_	0.075

The first set of 26 analogs we synthesized was tested *in vitro* and we observed that amides with chain lengths of three carbons or less were the most active (see Table 4). The incorporation of a hydroxyl group on the amide generally increased potency (MLS-0146161 and MLS-0146165). Branching of the amides generally decreased potency in our assay, especially when the chain length was greater than three carbon atoms (data not shown). We synthesized one hydrazide analog (MLS-0146145) and it was very potent. Substitution on the phenyl ring was limited to the 2,4-dichloro and 2,4-dichloro-5-fluoro- substituent pattern. In all cases the 2,4-dichloro analogs were more potent than the corresponding 2,4-dichloro-5-fluoro analogs. In light of these results we synthesized a new set of 28 pyrazole analogs that served to investigate several portions of the pyrazole structure. 

When exploring the SAR when the substituent pattern on the aromatic ring was changed while fixing the amide as the 2-hydroxy ethyl moiety of MLS-0146161, we found the best compound in this series was the 2,3,4-tri-Cl-phenyl analog MLS-0146291. It shows exceptional activity with an IC_50_ of 5.19 nM. This is the compound that we nominate as our probe. Other analogs synthesized increase diversity of the hydrazides and introduce more hydroxy groups on the amide tails while fixing the 2,4-dichloro substituents on the aromatic ring. In this series, when the hydroxyl ethyl chain was increased by one or two additional carbon atoms the activity did not decrease. When a bis-hydroxyl ethyl side chain was introduced the activity remained the same as MLS-0146161.

**Figure 7:**
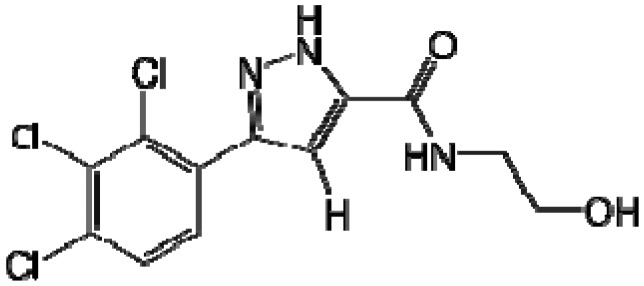
Highly potent TNAP inhibitor probe MLS-0146291 IC_50_ = 5.19 nM.

Compound MLS-0146291 was tested in assays against another alkaline phosphatase, PLAP, which shares 50% sequence identity with TNAP, and a housekeeping enzyme (GAPDH). The compound did not show any inhibition at or below 10 µM in these assays providing a >2,000-fold selectivity index against the closest counter-targets. In addition, the lead pyrazole CID-646303 has been extensively profiled in assays deposited in PubChem. Out of 201 assays tested the compound was inactive in 199 and inconclusive in two assays.

*Triazole series.* Most of the triazole derivatives (Figure 2) identified from the primary screen belong to a more specific thio-phenyl-triazole subseries, resembling the ones identified in a previous screening effort [11]. A triazole derivative MLS-0067142 (Table 3) was the most potent in the series but was a singleton, so insufficient to derive a nascent SAR. It is interesting that in several of the hits of the previous bisarylsulfonamide series [44] the presence of a pyridine moiety appeared to be a tight requirement (right-hand aryl group in Figure 3). The triazole moiety may be acting as a substitute for the pyridine ring.

### 2.6. Weak TNAP Inhibitory Triazoles Scaffolds Serendipitously are More Selective for Intestinal Alkaline Phosphatase (IAP) with Compound with a Novel Mode of Inhibition

We originally developed the luminescence-based IAP assay (PubChem AID 1017) for the selectivity characterization of potential TNAP inhibitor probes. A similar assay developed at the *CPCCG* to screen for TNAP HTS led to identification of several potent scaffolds that were completely inactive in the TNAP colorimetric assay provided by the assay provider. One of these scaffolds is characterized in detail below as a selective IAP inhibitor (Table 7).

Representatives of the leading active and inactive TNAP inhibitor structural series were then counter-screened against the IAP assay. It was observed that unlike the other scaffolds, some triazole derivatives potently inhibit the IAP isozyme. Expanded screening (77 compounds total) led to identification of a compound that is a selective *nanomolar* inhibitor of IAP, CID-296732.

**Table 7:** SAR and Selectivity of phtalimide triazole inhibitor series. 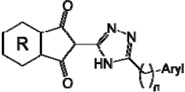

Compound	Fused-Ring (R =)	n	Aryl	IC_50_ µM	
				TNAP- Lumi	IAP-Lumi
CID-2975791	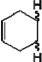	1	Phenyl	0.21	0.54
CID-296732	3-Cyclohexenyl	0	Phenyl	0.80	0.12
MLS-0091978	Phenyl	1	Phenyl	0.67	8.87
MLS-0091981	Cyclohexanyl	1	Phenyl	1.57	1.10
MLS-0091987	3-Cyclohexenyl	2	Phenyl	27.9	0.72
MLS-0091984	Cyclohexanyl	2	Phenyl	>100	1.75
MLS-0091982	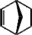	2	Phenyl	30.6	4.75
MLS-0091980	3-Cyclohexenyl	0	H	>100	>100
MLS-0091977	3-Cyclohexenyl	0	3-Pyridinyl	10.5	1.77
MLS-0091966	3-Cyclohexenyl	0	2-Furanyl	4.68	0.41
MLS-0010757	3-Cyclohexenyl	0	Phenyl	>100	>100

**Figure 8:**
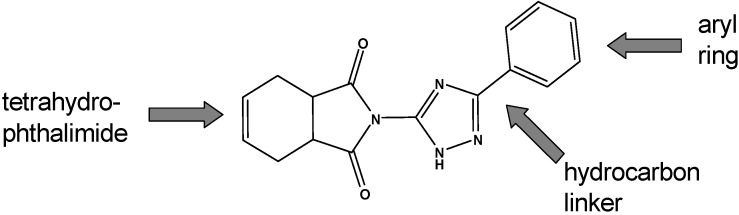
Points of variation for SAR of CID92946732.

An additional sub-micromolar IAP assay using *p*-nitrophenol phosphate (pNPP) as substrate was developed at the *CPCCG* and was utilized for hit confirmation. Lead compounds were tested in a panel of assays consisting of: (1) TNAP luminescent assay (substrate: CDP-star); (2) TNAP colorimetric assay (substrate: pNPP); (3) PLAP luminescent assay (substrate: CDP-star); (4) IAP luminescent assay (substrate: CDP-star); (5) IAP colorimetric assay (substrate: pNPP); (6) solubility and (7) glyceraldehyde-3-phosphate dehydrogenase (GAPDH) assay. The latter two assays are designed to identify compounds that are likely to interfere with the assay components and would appear as non-specific inhibitors. Our approach to exploring the SAR for the IAP inhibitor series is shown graphically in Figure 8. 

For initial SAR studies we focused on three structural elements in the molecule to investigate: (a) the tetrahydrophthalimide ring; (b) the aryl ring; (c) the hydrocarbon linker between the aryl ring and the triazole unit. To date, 61 analogs in the triazole series have been purchased and tested, while a further 11 analogs have been synthesized. Compounds were characterized in the luminescence-based assay (IAP-L, see Table 5) to determine their potency against IAP. Inhibition of TNAP (TNAP-L) by analogs was determined in parallel for selectivity profiling. The *in vitro* data for this series suggest that there is a tractable SAR emerging for the IAP inhibitor triazole derivatives. 

The original hit for this series (CID-2975791) was identified from HTS as a potent inhibitor of TNAP. Selectivity profiling revealed this compound also to be a submicromolar inhibitor of IAP (Table 2). The phthalimide analog (MLS-0091978) was less potent against IAP (8.9 µM) and more potent against TNAP (0.67 µM), while the hexahydrophthalimide was equipotent against both enzymes. However, extension of the linker chain length to ethyl gave a compound (MLS-0091987) with good potency against IAP (0.72 µM) and 40-fold selectivity over TNAP. Two other analogs with ethyl linkers (MLS-0091984 and 0091982) show similarly high selectivity for IAP, but are not as potent. Removal of the benzyl moiety in the hit, as in MLS-0091980, leads to elimination of activity against both isozymes, as does amine substitution onto the triazole ring (MLS-0010757). On the other hand, removal of just the methylene linker in the hit to the phenyl triazole derivative gives a compound (CID-2946732) that has an IC_50_ = 0.12 µM *vs.* IAP and is 7-fold selective for IAP over TNAP. Furthermore, replacement of the phenyl moiety with pyridine (MLS-0091977) or furan (MLS-0091966) gives derivatives with 6- and 10-fold selectivity, respectively, for IAP *vs.* TNAP, and in the case of the furan derivative MLS-0091966 IC_50_ = 0.41 µM. Taking all the data into consideration the phenyl triazole derivative CID-2946732 was selected as a probe for IAP. It should be noted that other analogs in the series, such as MLS-0091987 and MLS-0091966 are also strong probe candidates, and work is ongoing to perform additional profiling studies to characterize these and other compounds in the series.

CID-2946732 demonstrated potent inhibition for IAP and selectivity *vs.* other alkaline phosphatase isozymes, TNAP and PLAP (Table 8). *PubChem* searches revealed that CID-2946732 was inactive in 68 other assays and demonstrated activity only in two additional assays, both for CYP isozymes: CYP2C9 and CYP2C19. These data suggest that CID-2946732 could serve as a valuable tool for the characterization of IAP biological function, especially in the gastrointestinal tract, where alkaline phosphatase activity is predominantly accounted for by the IAP isozyme.

**Table 8:** Selectivity profile for IAP probe CID-2946732.

CID	Structure	Compound ID	IAP-Lumi	IAP-Color	TNAP-Lumi	TNAP-Color	PLAP-Lumi	2C19	2C9
2946732	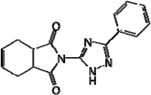	MLS-0091968	0.122	1.38	0.795	>100	4.81	79.6% I @	92.5% I @
			(n=7)	(n=7)	(n=7)	(n=6)	(n=3)	5 µM	5 µM

This compound has favorable calculated properties, low molecular weight (294.3) and structural features that are similar to known drugs, and can thus be considered a “drug-like” molecule. It has a log P value of 2.096, with 1–3 being the range of most oral drugs. The total polar surface area (TPSA) is 78.96, also indicative of good oral bioavailability. It is also a rigid molecule with two rotatable bonds, one H-bond donor and six H-bond acceptors. Thus, this probe’s physicochemical properties make it useful for continued study, including *in vivo* models of efficacy and tolerance.

The compound CID-2946732 inhibited IAP in two assays that utilize different substrates and modes of detection. The compound is soluble below 25 µM and demonstrated solubility issues above 25 µM. It had no effect on GAPDH activity and demonstrated no cytotoxicity in multiple assays deposited in *PubChem*. CID-2946732 is a versatile, novel Chemical Probe for the characterization of IAP biological activity in various biological systems. Its potency by far (>6,500-fold) and selectivity exceeds all other existing small-molecule inhibitors of IAP. Mechanistic studies performed on the CID-2946732 demonstrated that it inhibits IAP non-competitive to the CDP-star substrate. This is a novel mode of inhibition, since all known Probes mentioned above are uncompetitive *vs.* the phosphate-donor substrate.

### 2.7. Identification and Optimization of PLAP-specific Chemical Probes from Catechol Series

**Figure 9:**
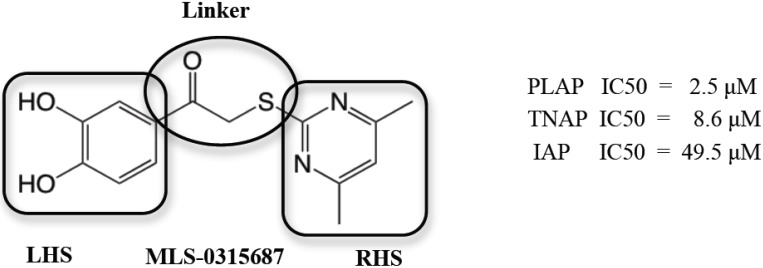
Depiction of the three regions of MLS-0315687 for SAR: Left Hand Side (LHS), Right Hand Side (RHS) and Linker region (encircled).

In collaboration with researchers at the Human Biomolecular Research Institute (HBRI), we developed a series of 3,4-dihydroxy substituted catechols that maintained the inhibitory potency of an original screening hit, MLS-0315687, as an inhibitor of PLAP. But through a series of molecular modifications on the “right and left hand sides” and the “linker” portions (Figure 9), PLAP inhibitors with greater inhibition selectivity over two other related isozymes, TNAP and IAP, were achieved [46]. 

**Figure 10:**
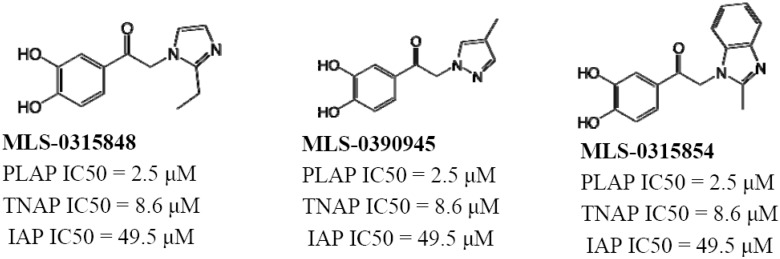
Example of Right Hand Side modifications of MLS-0315687.

MLS-0315848, possessing a 2-ethylimidazole substituent, was over 27-fold more selective as an inhibitor of PLAP compared to TNAP and IAP, while maintaining a reasonable PLAP inhibitory potency (IC_50_ = 4.2 μM). MLS-0390945 was more than 50- and 25-fold selective as an inhibitor of PLAP over TNAP and IAP, respectively (see below). Finally, MLS-0315854 was 10- and 40-fold more selective inhibitor of PLAP over IAP and TNAP, respectively. All three compounds were more potent inhibitors of PLAP than previously reported isozyme-selective inhibitors of APs (Table 1) and considerably more selective. These Right Hand Side (RHS) modifications improved upon the selectivity of the screening hit, MLS-0315687, especially with regard to TNAP selectivity (Figure 10). 

**Figure 11:**
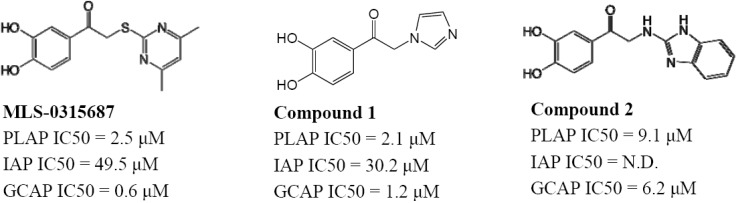
PLAP selective compounds with additional selectivity over GCAP .

MLS-0315687 and two related compounds are also somewhat selective for PLAP over GCAP in the single digit micromolar range (Figure 11). Because of their inhibitory selectivity, these catechol compounds may be useful as tools to understand the physiological role of PLAP in biology and pharmacology and may have applications for treating cancer or in cancer diagnostics because elevated levels of TNAP are found in ovarian and testicular cancer patients.

### 2.7. TNAP Activators are Detected with the Improved TNAP Assay

The CDP-Star TNAP assay was also sensitive enough, and possessed a wide enough dynamic range, to allow detection of potential activators of TNAP under the same conditions as optimized for the inhibition assay. For the TNAP activator project we used an assay with no DEA in the reaction mix (AID 1001). This would have been impossible with the colorimetric assay, which does not have sufficient signal with low DEA and would have required the use of much higher TNAP level (100-fold more). For this assay, 195,565 compounds were tested at 20 µM/compound and 150 compounds had activity ≥3 µM. The average Z’ for the screen was 0.77; the signal to background was 31.8; the signal to noise was 295 and the signal to window was 11.8. Of the primary hits, 145 compounds out of 150 were available from the MLSMR for DMSO dose-response confirmation. Of the primary hits, 42 were confirmed and dry powder samples were purchased from the vendors for reconfirmation. Two scaffolds, a spirocyclic derivative and a triazolecarboxamide, were identified as TNAP activators. These are currently being pursued to define their full SAR, mechanisms of action, and pharmacological profiles.

## 3. Experimental Section

### 3.1. Reagents

The CDP-star substrate (2-chloro-5-(4-methoxyspiro{1,2-dioxetane-3,2′(5′-chloro)-tricyclo-[3.3.1. 13.7]-decan}-4-yl)-1-phenyl phosphate disodium salt; CAS 160081-61-9) was obtained from New England Biolabs (Ipswich, MA). All other chemicals were of an analytical grade and obtained from Sigma Aldrich.

### 3.2. Compound Collection Used in HTS

The National Institutes of Health (NIH) Molecular Libraries Small Molecule Repository (MLSMR, http://www.mli.nih.gov/mlsmr) supplied the compound library. The MLSMR, funded by the NIH, is responsible for the selection of small molecules for HTS screening, their purchase and quality control (QC) analysis, library maintenance, and distribution within the NIH Molecular Libraries Screening Center Network (MLSCN, http://www.mli.nih.gov/mlscn). Both MLSMR and MLSCN are parts of the Molecular Libraries Initiatives (MLI, http://nihroadmap.nih.gov/molecularlibraries) under the NIH Roadmap Initiatives (www.nihroadmap.nih.gov). MLSMR compounds are acquired from commercial and in part from academic and government sources and are selected based on the following criteria: samples are available for resupply in 10-mg quantity, are at least 90% pure, have acceptable physicochemical properties, and contain no functional groups or moieties that are known to generate artifacts in HTS (http://mlsmr.glpg.com/). Compounds are selected to represent diversified chemical space with clusters of closely related analogs around them to aid in the HTS-based structure-activity relationship (SAR) analysis. All positives were also tested in an assay panel containing: (1) TNAP colorimetric assay (substrate: *p*-nitrophenol phosphate); (2) placental alkaline phosphatase (PLAP) luminescent assay (substrate: CDP-star); (3) intestinal alkaline phosphatase (IAP) luminescent assay (substrate: CDP-star); (4) solubility and (5) glyceraldehyde-3-phosphate dehydrogenase (GAPDH) assay. The latter two assays are designed to identify compounds that are likely to interfere with the assay components and would appear as unspecific inhibitors.

### 3.3. Expression and Preparation of Test Enzymes

*TNAP, PLAP and IAP*: Expression plasmids containing a secreted epitope-tagged TNAP, PLAP or IAP were transfected into COS-1 cells for transient expression [39,40,47]. The medium was replaced with Opti-MEM 24 h later, and the serum-free media containing secreted proteins were collected 60 h after electroporation. The conditioned medium was dialyzed against Tris-buffered saline (TBS) containing 1 mM MgCl_2_ and 20 mM ZnCl_2_ (to remove phosphate) and filtered through a 0.22-μm cellulose acetate filter.

### 3.4. Inhibition Assays for AP Isozymes

The colorimetric TNAP assay was performed as previously described [11] with slight modifications. TNAP in assay buffer containing 1M DEA-HCl (pH 9.8), 1 mM MgCl_2_, and 20 µM ZnCl_2_ was added with 0.5 mM pNPP substrate, and after an 1 hour incubation, the reaction was terminated by adding 2,3-dimercapto-1-propane sulfonate (a compound that binds Zn^2+^ with high affinity, thus inactivating the enzyme) to 100 μM. TNAP activity was measured using absorbance at 405 nm (OD405assay) or at 620 nm after addition of malachite green–based phosphate detection reagent (MG assay) prepared according to a published procedure [48].

Development of the novel luminescent TNAP assay using CDP-star substrate and detailed protocol of its application for HTS is described in more detail elsewhere [41,43]. Also a detailed protocol of this assay can be found in PubChem AID 518. Briefly, the assay was 50 μM CDP-star substrate in assay buffer containing 100 mM DEA-HCl (pH 9.8), 1 mM MgCl_2_, and 20 μM ZnCl_2_. The luminescence signal was measured after a 30-min incubation at room temperature on an EnVision plate reader (PerkinElmer, Waltham, MA). Analogous luminescent assays were optimized and used for to screen for inhibitors and support SAR studies for PLAP (PubChem AID 1512) and IAP (PubChem AID 1017) with the CDP-star concentration adjusted to their respective K_m_ values, 85 μM and 177 μM, respectively. Detailed protocols can be found in the respective AIDs, however, the final buffer conditions were 100 mM DEA, pH 9.8, 1.0 mM MgCl_2_, 0.02 mM ZnCl_2_ for both PLAP and IAP.

The pyrophosphatase (PP_i_ase) activity of TNAP was assayed discontinuously by measuring the amount of inorganic phosphate liberated, according to the procedure used previously [49] adjusting the assay medium to a final volume of 0.5 mL. Standard assay conditions were 50 mmol/L Tris buffer, pH 7.4, containing 2 mmol/L MgCl_2_ and substrate. The reaction was initiated by the addition of the enzyme and stopped with 0.25 mL of cold 30 %TCA at appropriate time intervals. The reaction mixture was centrifuged at 4,000× g, and phosphate was quantified in the supernatant after pH neutralization with 0.1 mol/L NaOH as described before [50]. 

### 3.5. Assays for AP Isozyme Activators

Additionally, the format and calculations used for TNAP activators can be found in PubChem AIDs 813 and 101, 1136, and 1659. Final buffer conditions were 50 mM CAPS, pH 9.8, 1.0 mM MgCl_2_, 0.02 mM ZnCl_2,_ with 100 µM CDP Star (near it’s K_m_) and either no or 100 mM DEA. 

### 3.6. HTS and Chemical Probe Development

Primary TNAP screening was completed on MLSMR 64,394 compounds using the luminescent assay at a single concentration of 20 μM. Levamisole at 1 mM concentration was used as positive control. A Z′ factor, a measure of assay quality was calculated [51] and found to be ~0.8. Following hit confirmation and chemical clustering, fresh powders of compounds and commercially available analogs were obtained for reconfirmation, isozyme selectivity, and initial SAR elucidation. Based on the nascent SAR, additional analogs were obtained commercially or by *de novo* design and synthesis, to improve potency and selectivity. Ultimately a best optimized compound was nominated as a *bonafide* chemical probe. Completed Chemical Probe Reports have been filed with the NIH Roadmap, Molecular Libraries Program Team, for review and approval.

## 4. Conclusions

It is often underappreciated how critical the configuration, particular experimental conditions, and signal generation and detection technologies employed during the development of a high-throughput assay (HTS) is to the outcome of a screening campaign. For example, fruitless screening campaigns for non-ATP competitive inhibitors of kinases using tracer levels of radioactive ATP and very low total ATP concentrations. More surprisingly, recently published comparisons of hits from an HTS screen against a nuclear receptor, do not support the common assumption that the same hits would be obtained regardless of assay technologies used [52]. In fact, of the 104, 23 and 57 compounds that were identified from AlphaScreen™, TRF and TR-FRET HTS formats for the same target, respectively, only 18 compounds were active in all three formats. A follow-up study to delineate the effect of the inherent assay variability in the primary screen on the difference in identified hits between assay formats was completed using a set of compounds in four separate runs in single-point determinations on the same nuclear receptor. The activity distributions using either the AlphaScreen™ or TR-FRET assay technologies yielded significantly different profiles [53]. A further detailed study of quadruplicate HTS replicates of the same target allowed a quantitative estimate of effect of assay variability on hit selection. These results further support the critical importance of the assay technology chosen on the selection of hits. In another comparison of the sensitivity and dose-response curves with respect to 384-well assay formats for tyrosine kinases [54], it was found that scintillation proximity assays (SPA) required the most enzyme (200 ng/well] for assay robustness, while fluorescence polarization (FP) required less (4 ng/well) and fluorescence resonance energy transfer (FRET) required the least (1 ng/well). Though the IC_50_ determined for a reference non-selective kinase inhibitor were comparable among these three formats, screening of a large library yielded different sets of hits [54].

Colorimetric alkaline phosphatase assays have been the gold standard for more than 20 years, especially in the clinical laboratory setting. It is also well known that fluorogenic assays are 10-fold more sensitive than colorimetric assays [55]. As fluorescent chemosensors and indicators have been developed [56] as well as offered as commercial kits from some of the most diversified life science reagents and kit providers. During the course of a MLSCN screening campaign for inhibitors of TNAP we adapted a classic colorimetric assay [11] and used it to screen a 20,500 member small molecule collection and obtained no hits, even when them substrates were set near their K_m_. We then developed a luminescent assay whose increased sensitivity and 1,000-fold increased dynamic range allowed robust measurable rates to be obtained at low and even in the absence of exogenously added aminoalcohols as terminal phosphate acceptors, usually necessary for AP reactions. Two clear pragmatic benefits of increased activity even at low amino alcohol concentrations are the need for much less TNAP enzyme. Reduced enzyme concentrations can often make or break the ability to complete a full HTS campaign. Also the limit of inhibitor potency (K_i_) that can be theoretically detected is equal to ½ [E]_tot_. Moreover, use of the aminoalcohols to accelerate the reaction and yield a larger signal also ensures that the alkaline phosphatases are working close to their maximal rates, and this makes the detection of small molecules that further activate the phosphatase difficult under those assay conditions. The luminescent assay designed allows us to use the same assay conditions to find both inhibitors and activators of TNAP. Activators of TNAP function may be useful for the treatment of milder forms of hypophosphatasia, as the therapeutic use of TNAP activators might sufficiently enhance residual TNAP activity to help resolve or prevent the rickets/osteomalacia characteristic of the disease [8]. 

A common feature of competitive inhibitors is that they are ineffective in the presence of elevated (relative to K_m_) concentrations of the corresponding substrate. As a consequence, chemical probes with this attribute could act as sensors of the local concentration of natural substrates of TNAP in a specific biological environment. Similarly, if employed as therapeutic agents, they have the potential to be selective with respect to specific physiological functions of TNAP. All known-to-date inhibitors of TNAP demonstrated uncompetitive mechanism with respect to phosphate-donating substrates. This improved assay has already demonstrated that it can find alkaline phosphatase inhibitors with diverse modes of action. In this example of screening for TNAP, the impact of this improved assay is the rescue from an apparently “failed” screen, three new classes of inhibitors. These would have been completely missed without the reconfiguration of the assay to allow more sensitive detection, as brought into focus by Figure 2 above. It has historically been difficult to find PLAP and IAP selective alkaline phosphatase inhibitors, as these isozymes are much closer in sequence similarity than compared to TNAP. The bisaryl sulfonamides and pyrazoles yielded two separate chemotypes with robust selectivity for TNAP over PLAP and IAP, with the sulfonamides demonstrating activity in cell based and *ex vivo* models of calcification, and the pyrazoles yielding exemplars of chemical probes with single digit nanomolar potency. But it must be emphasized that the improved assay allowed us to uncover the triazole scaffold series that yielded both PLAP and IAP selective inhibitor probes, and novel TNAP activators. The triazole activator had modest potency (~5 µM EC_50_) but showed activity in the cell-based model and appears to be uncompetitive with DEA. The assay also uncovered a second scaffold with TNAP activator properties at about 20-fold higher potency (0.2 µM EC_50_) for TNAP and that is competitive with DEA.

To date, the only known classes of alkaline phosphatase activators are hydroxyl-containing compounds, such as diethanolamine (DEA), that act as a phosphoacceptor substrate and exhibit effects in the high mM concentration range. Compounds with a similar mode of action are expected to be competitive with DEA and demonstrate diminished stimulating potential if tested in the presence of high DEA concentrations. On the other hand, one could potentially envision other venues for TNAP activity acceleration, such as influencing the hydrogen-bonding pattern within, or in the vicinity of, the TNAP active site observed with some TNAP mutants or even allosteric interactions between active sites of the TNAP dimer. These effectors may not necessarily be competitive with DEA. Therefore a secondary goal was to identify different groups of hits and classify them according to their mode of action with respect of phospho-acceptor concentration. The current improved luminescent assay will be a critical tool for these studies and explorations, as well as the ready panel of AP selectivity assays in the same commensurate luminescence format. 
